# Synaptic configuration and reconfiguration in the neocortex are spatiotemporally selective

**DOI:** 10.1007/s12565-023-00743-5

**Published:** 2023-10-14

**Authors:** Jaerin Sohn

**Affiliations:** Department of Systematic Anatomy and Neurobiology, Graduate School of Dentistry, Osaka University, Suita, Osaka 565-0871 Japan

**Keywords:** Cell type, Neocortex, Neural circuit, Plasticity, Synapse

## Abstract

Brain computation relies on the neural networks. Neurons extend the neurites such as dendrites and axons, and the contacts of these neurites that form chemical synapses are the biological basis of signal transmissions in the central nervous system. Individual neuronal outputs can influence the other neurons within the range of the axonal spread, while the activities of single neurons can be affected by the afferents in their somatodendritic fields. The morphological profile, therefore, binds the functional role each neuron can play. In addition, synaptic connectivity among neurons displays preference based on the characteristics of presynaptic and postsynaptic neurons. Here, the author reviews the “spatial” and “temporal” connection selectivity in the neocortex. The histological description of the neocortical circuitry depends primarily on the classification of cell types, and the development of gene engineering techniques allows the cell type-specific visualization of dendrites and axons as well as somata. Using genetic labeling of particular cell populations combined with immunohistochemistry and imaging at a subcellular spatial resolution, we revealed the “spatial selectivity” of cortical wirings in which synapses are non-uniformly distributed on the subcellular somatodendritic domains in a presynaptic cell type-specific manner. In addition, cortical synaptic dynamics in learning exhibit presynaptic cell type-dependent “temporal selectivity”: corticocortical synapses appear only transiently during the learning phase, while learning-induced new thalamocortical synapses persist, indicating that distinct circuits may supervise learning-specific ephemeral synapse and memory-specific immortal synapse formation. The selectivity of spatial configuration and temporal reconfiguration in the neural circuitry may govern diverse functions in the neocortex.

## Introduction

One of the fundamental characteristics in the brain is the synaptic connections among various neurons. Neuronal signal outputs can influence the activity of a finite neuronal population via chemical synapses that are formed in contact sites between them. Neurons spread neurites such as dendrites and axons, and this morphological property increases the surface area and the chance of contacts, resulting in thousands of synaptic inputs to single neurons. Dendrites and axons of neurons do not simply radiate in all directions from their somata; rather, individual neurons display unique ramification patterns (Markram et al. 2004; Xu et al. 2006; Ascoli et al. 2008; Uematsu et al. 2008; Kubota 2014; Feldmeyer et al. 2018; Gouwens et al. 2020). Since this diversity of neuronal morphologies would reflect the functional properties of individual cell populations, the wiring diagrams composed of the synaptic connections should be depicted based on the presynaptic and postsynaptic identities of highly diverse neurons.

**Fig. 1:**
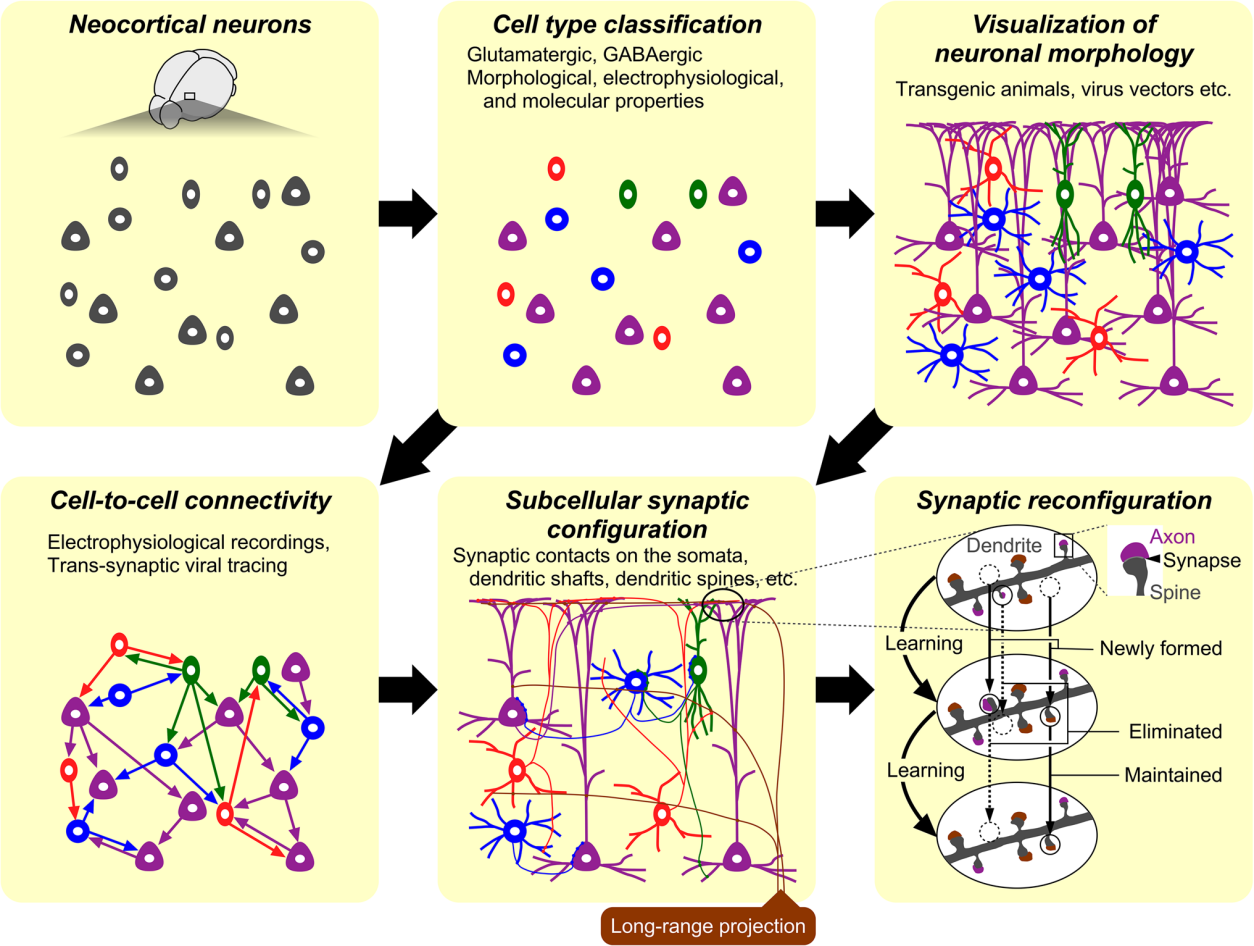
Roadmap for depicting spatiotemporal selectivity of synaptic configuration and reconfiguration. Depicting the diagram of the neocortical network requires cell type classification. Connectivity among cell subtypes at a cellular level can be recorded by electrophysiology. Observation of synaptic configuration at a subcellular resolution needs visualization of somatodendritic and axonal morphology of individual neurons. The subcellular synaptic layout can be rearranged by learning, showing formation, maintenance and elimination of synapses

Since Cajal (1911) and Lorente de Nó (1938) described qualitative wiring diagrams, the architecture of neural circuits in the neocortex has been quantified through the developments of neuronal labeling techniques such as immunohistochemistry, neuronal tracers, virus vectors and transgenic animals. The morphological characteristics of neurons well correspond to the intrinsic molecular and electrophysiological properties (Markram et al. 2004; Xu et al. 2006; Ascoli et al. 2008; Kubota 2014), indicating that the functional roles neurons can play depend on these cell type profiles. Therefore, the quantitative wiring diagram of the neocortex primarily requires the classification of neuronal subtypes (Fig. 1). In addition, the development of gene engineering techniques allows the cell type-specific visualization of the entire somatodendritic and axonal arborization (Fig. 1). The classification and labeling tools have expedited the anatomical investigation of the wiring principles in the neocortex.

Cell-to-cell connections can be identified by electrophysiological recordings (Fig. 1). Virus vectors such as herpes simplex virus (Zhang et al. 2015), rabies virus (Kelly and Strick 2000), vesicular stomatitis virus (van den Pol et al. 2009), or adeno-associated virus (AAV) (Zingg et al. 2017) may be applicable for trans-synaptic tracings, though the mechanism and specificity of trans-synaptic transmission remain hypothetical and controversial with potential caveats (Beier 2019). High-resolution microscopy such as electron microscopy verifies the presence of synaptic connections, and anatomical observation at a synaptic spatial resolution provides the information of the subcellular synaptic layout (Fig. 1). For instance, the subcellular distribution of γ-aminobutyric acid-ergic (GABAergic) inputs in glutamatergic pyramidal cells has been well established (Somogyi et al. 1998; Thomson and Bannister 2003; Jiang et al. 2013; Kubota 2014; Kubota et al. 2015, 2016): parvalbumin-expressing (PV^+^) basket cells with a fast-spiking firing property, one of the major GABAergic neuronal populations, are reported to largely innervate the perisomatic domain of pyramidal cells, while the other GABAergic cell types such as somatostatin-expressing (SOM^+^) neurons preferentially target the dendrites. Likewise, glutamatergic and GABAergic synaptic convergence in GABAergic neurons also shows compartmentalized subcellular innervation in a presynaptic cell type-dependent manner (Kameda et al. 2012; Hioki et al. 2013, 2018; Sohn et al. 2016). This spatial selectivity of subcellular innervation patterns indicates presynaptic cell type-specific regulation of single neuron excitability.

Furthermore, synaptic connections are not static but dynamic, and synaptic plasticity is believed to be the cellular basis of learning. In the primary motor cortex (M1), motor learning triggers synaptogenesis in the apical tuft of layer (L) 5 pyramidal cells (Xu et al. 2009; Yang et al. 2009; Wang et al. 2011; Fu et al. 2012). The apical tufts of pyramidal cell dendrites are located in L1, and excitatory afferents in L1 of the neocortex consist of both cortical and thalamic axon fibers (Kuramoto et al. 2009, 2015; Kaneko 2013; Ueta et al. 2014; Shigematsu et al. 2016; Hasegawa et al. 2020). We showed that the neighboring corticocortical and thalamocortical synapses display unique dynamics in motor skill learning (Sohn et al. 2022). Identification of presynaptic cell types combined with observation of synaptic dynamics in vivo demonstrates that the synaptic dynamics during learning depend on the origin of presynaptic axons innervating newly generated synapses (Fig. 1). This temporally selective reconfiguration of cortical synapses indicates that corticocortical and thalamocortical synapses play distinct functional roles in learning and memory.

In this review, the author summarizes the spatiotemporal selectivity of the neocortical synaptic wirings. We have anatomically investigated the wiring diagram, “connectome”, composed of diverse cell types in the neocortex, and we have revealed the spatial selectivity of the subcellular synaptic configuration in single cells with fixed brain samples. This neural circuit map of the neocortex, however, lacks the information about the role that each wiring plays in a particular function of animals. We further tackled the issue of how the presynaptic identity might determine the rewiring process in skill learning. Observation of circuit-specific synaptic dynamics may allow us to unveil “functional connectome” involved in animal’s behavior in vivo.

## Cell type classification in the neocortex

The neocortex contains a variety of cell types, and the connection selectivity in the neural circuit notably depends on the cell characteristics of presynaptic and postsynaptic neurons. Neocortical neurons can be largely classified into glutamatergic excitatory neurons (~ 80%) and GABAergic inhibitory neurons (~ 20%). Although glutamatergic pyramidal cells abound in morphological diversity (van Aerde and Feldmeyer 2015), they are primarily classified by the laminar location of their somata. The laminar location roughly corresponds to their typical projection targets (Molyneaux et al. 2007; Petreanu et al. 2007; Mao et al. 2011; Han et al. 2018): for example, L2/3 pyramidal cell axons arborize within the telencephalic regions [intratelencephalic (IT) cells], while L5 of the frontal cortex consists of two major subtypes, contralaterally projecting IT cells and pontine nuclei-projecting pyramidal tract (PT) cells (Hallman et al. 1988; Cowan and Wilson 1994; Reiner et al. 2003; Arlotta et al. 2005; Morishima and Kawaguchi 2006; Morishima et al. 2011; Ueta et al. 2014; Kawaguchi 2017). L5 IT cells in the secondary motor cortex (M2) broadly send feedback signals to multiple cortical areas, and contain diverse projection types with different L1-targeting preference (Im et al. 2023). PT cells project to the ipsilateral pontine nuclei (Kita and Kita 2012), and they also innervate the other brain regions outside the telencephalon such as the thalamus, superior colliculus, zona incerta, the other brainstem areas and the spinal cord (Hirai et al. 2012; Kita and Kita 2012). PT cells are responsible for motor commands and preparatory activities, and could further be divided into subclasses by the difference of the major target subcortical regions such as the thalamus and the brainstem (Economo et al. 2018). L5 PT cells in the motor cortex are responsible for oscillatory activity and motor learning compared with IT cells (Otsuka and Kawaguchi 2021).

L2/3 pyramidal cells are also composed of subtypes according to their projection patterns (Little and Carter 2012; Lu et al. 2017; Liu and Carter 2018). For example, we identified two types of L2 pyramidal cells in M2 with different electrophysiological and morphological characteristics: those projecting to the contralateral M2 (cM2) and those projecting to the ipsilateral perirhinal cortex (iPRC) (Ueta et al. 2019). In addition, in the primary somatosensory barrel cortex (S1BF), L2/3 cells can be classified into neurons projecting to M1 and those projecting to the secondary somatosensory area (S2), exhibiting different membrane potential dynamics associated with tactile perception (Yamashita et al. 2013). Therefore, these target-dependent subdivisions may reflect functional diversity among L2/3 cells.

GABAergic cells are also morphologically and electrophysiologically heterogeneous. The morphological and electrophysiological identities well correspond to the gene expression such as calcium-binding proteins and neuropeptides, and their molecular characteristics are utilized for identification of the subpopulations (Kubota et al. 1994, 2011; Kawaguchi and Kubota 1996; Gonchar and Burkhalter 1997; Xu et al. 2006, 2010; Uematsu et al. 2008; Rudy et al. 2011; Hioki et al. 2013; Pfeffer et al. 2013; Kepecs and Fishell 2014; Gouwens et al. 2020). In the mouse neocortex, PV^+^ and SOM^+^ neurons account for approximately ~ 40% and ~ 30% of GABAergic neurons, respectively, and remaining ~ 30% of them express ionotropic serotonin receptor 5HT-3a (5HT-3aR) (Rudy et al. 2011; Tremblay et al. 2016; Feldmeyer et al. 2018). PV^+^ neurons include fast-spiking basket cells and chandelier cells, which innervate the perisomatic region of pyramidal cells, whereas the SOM^+^ population contains dendrite-targeting Martinotti cells. The 5HT-3aR^+^ subpopulation includes vasoactive intestinal polypeptide-expressing (VIP^+^) neurons with vertically oriented dendrites and axons, and non-VIP cells such as neurogliaform cells and L1 interneurons (Lee et al. 2010; Schuman et al. 2019). The significance of the classification based on these molecular profiles has been supported by the fact that individual GABAergic subclasses such as PV^+^, SOM^+^ and VIP^+^ neurons are involved in distinct functions in vivo (Gentet et al. 2012; Lee et al. 2012, 2019; Lovett-Barron et al. 2012; Wilson et al. 2012; Pi et al. 2013; Fu et al. 2014; Kepecs and Fishell 2014; Zhang et al. 2014; Kamigaki and Dan 2017; Abbas et al. 2018; Adler et al. 2019; Xu et al. 2019; Yu et al. 2019).

**Fig. 2:**
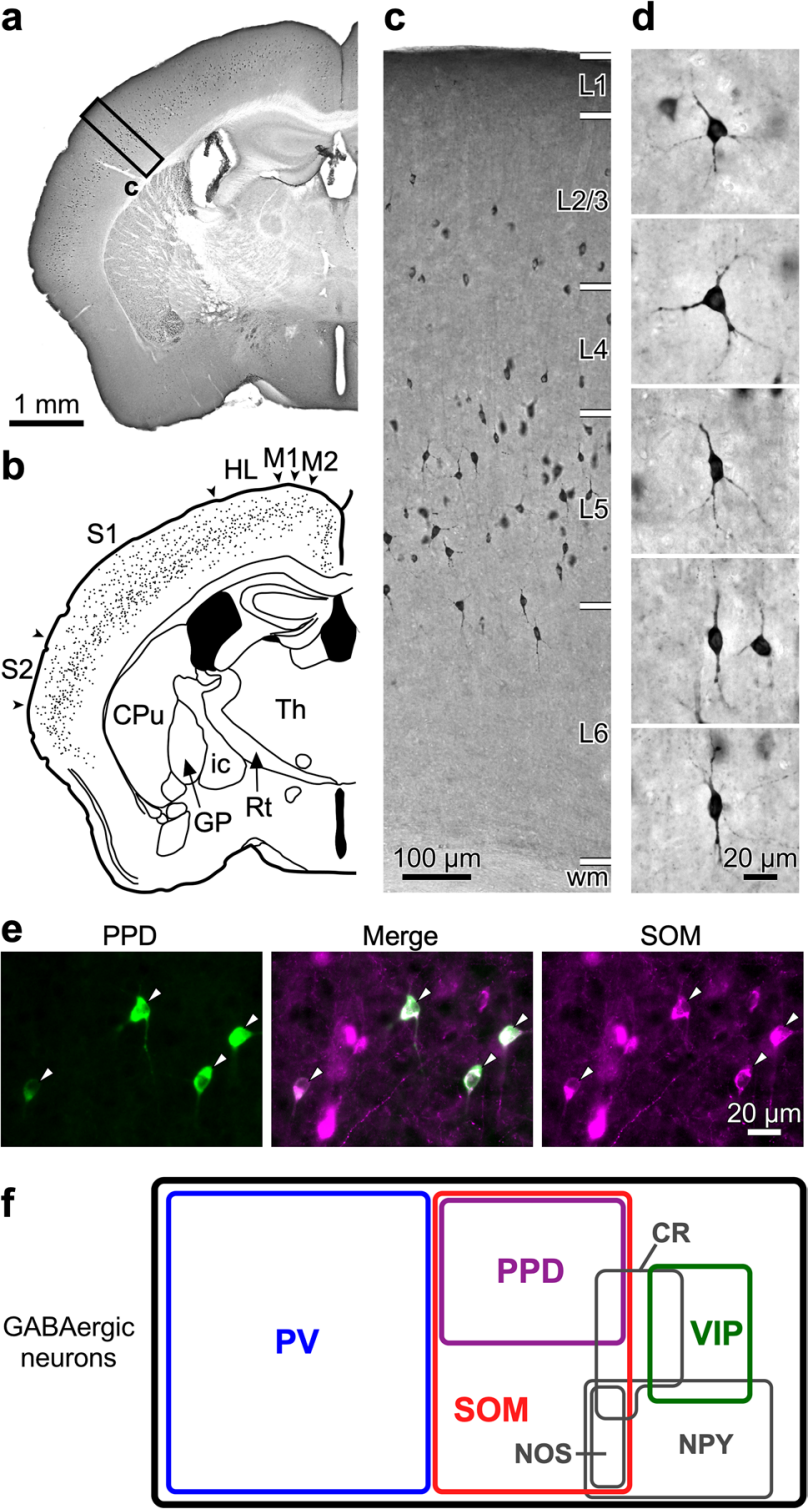
GABAergic neuronal subclasses and characterization of PPD^+^ cells in the neocortex. **a** Immunohistochemistry for PPD. **b** Distribution of PPD^+^ neurons in the neocortex. PPD^+^ cell bodies are plotted in a coronal brain section including S1BF. *CPu* caudate-putamen, *GP* globus pallidus, *HL* hindlimb area, *ic* internal capsule, *M1* primary motor area, *M2* secondary motor area, *Rt* thalamic reticular nucleus, *S1* primary somatosensory area, *S2* secondary somatosensory area, *Th* thalamic nuclei. **c** Magnified image of the vertical strip in panel **a**. wm, white mater. **d** Multipolar and bipolar somatodendritic morphology of PPD^+^ neurons. **e** Double immunofluorescence labeling for PPD (green) and SOM (magenta) in S1BF. Arrowheads indicate PPD^+^ cell bodies. **f** Drawing representing the proportion of GABAergic cell subtypes in the neocortex. *CR* calretinin, *NPY* neuropeptide Y, *NOS* neuronal nitric oxide synthase. Modified with permission from Sohn et al. (2014)

The GABAergic cell type taxonomy as above was reproducible by recent large-scale transcriptomic studies (Zeisel et al. 2015; Tasic et al. 2016; Gouwens et al. 2020; Sun et al. 2021) and also applicable to the human neocortex (Hodge et al. 2019). These datasets with systematic high-throughput measurements and analyses, however, have substantiated the further diversity in the previously defined cell subclasses. In fact, SOM^+^ axonal ramification patterns are diverse in S1BF (Tremblay et al. 2016; Nigro et al. 2018) and morphologically distinct SOM^+^ subtypes exhibit opposite activity changes that are dependent on the laminar locations and the brain state (Muñoz et al. 2017). In addition, SOM^+^ neurons are diverse in gene expression, which can be subdivided into calbindin-, calretinin-, neuropeptide Y-, neuronal nitric oxide synthase-, and/or nicotinic acetylcholine receptor α2 subunit-expressing neurons (Kubota et al. 1994; Kawaguchi and Kubota 1997; Ma et al. 2006; Xu et al. 2010; Tasic et al. 2016; Hilscher et al. 2017). We showed that preprodynorphin (PPD), the precursor of an opioid peptide dynorphin, was also expressed selectively in SOM^+^ neurons (Sohn et al. 2014) (Fig. 2), which is consistent with a transcriptomic study (Smith et al. 2019). PPD^+^ cells are distributed throughout the neocortex (Fig. 2a, b), mostly in L4 and L5 (Fig. 2c). Individual PPD^+^ neurons show multipolar or bipolar somatodendritic morphology (Fig. 2d). PPD-immunoreactive cell bodies co-express SOM (Fig. 2e), indicating SOM^+^ neurons can be divided into two subtypes by PPD expression (Fig. 2f). Intriguingly, a following study showed that the proportion of PPD^+^ cells in SOM^+^ neurons of S1BF can fluctuate dynamically during tactile information-associated learning (Loh et al. 2017), implying that the dynorphin expression level in the neocortex may represent a functional state of SOM^+^ neurons that are sensitive to developmental and environmental factors underlying human psychiatric disorders (Casello et al. 2022). The morphological, electrophysiological and functional properties of these diverse SOM^+^ cell subtypes will be further determined via future technical development (Hostetler et al. 2023).

## Gene engineering for visualization of neuronal morphology

Quantitative anatomical diagrams of synaptic wirings in the neocortex involve complete somatodendritic visualization of postsynaptic cells of interest as well as the information of the presynaptic cell identity. Gene engineering techniques are applicable for Golgi-like staining of particular neuronal subsets. Transgenic or bacterial artificial chromosome (BAC) transgenic mouse lines have been developed for brain-wide expression of fluorescent proteins in a cell type-specific manner (Feng et al. 2000; Ma et al. 2006; Kameda et al. 2012; Kaneko et al. 2018). In utero electroporation allows cell birthday-specific gene transduction in the brain region of interest (Saito and Nakatsuji 2001; Tabata and Nakajima 2001), which is applied for layer-selective cell labeling in the neocortex. Virus vectors, such as lentivirus (Naldini et al. 1996; Zufferey et al. 1997; Miyoshi et al. 1998; Amendola et al. 2005; Cronin et al. 2005; Hioki et al. 2007, 2009; Hirano et al. 2013; Kato and Kobayashi 2020), adenovirus (Niwa et al. 1991; Moriyoshi et al. 1996; Tamamaki et al. 2000; Tomioka and Rockland 2006), canine adenovirus-2 (Soudais et al. 2001; Junyent and Kremer 2015), rabies virus (Kelly and Strick 2000), Sindbis virus (Altman-Hamamdzic et al. 1997; Gwag et al. 1998; Furuta et al. 2001), and AAV (Kaplitt et al. 1994; Tervo et al. 2016; Challis et al. 2022), are available for anterograde and/or retrograde neuronal labeling by local transfection in the vicinity of injection sites (Davidson and Breakefield 2003; Callaway 2005). In addition, transgenic and knock-in mouse lines with expression of recombinases such as Cre and Flp (Taniguchi et al. 2011; Gerfen et al. 2013; Daigle et al. 2018) are employed for cell type-specific labeling in combination with reporter mice or virus vectors (Livet et al. 2007; Madisen et al. 2010; He et al. 2016; Daigle et al. 2018). The availability of these genetic tools has been accelerating the exploration of the neural network architecture.

**Fig. 3:**
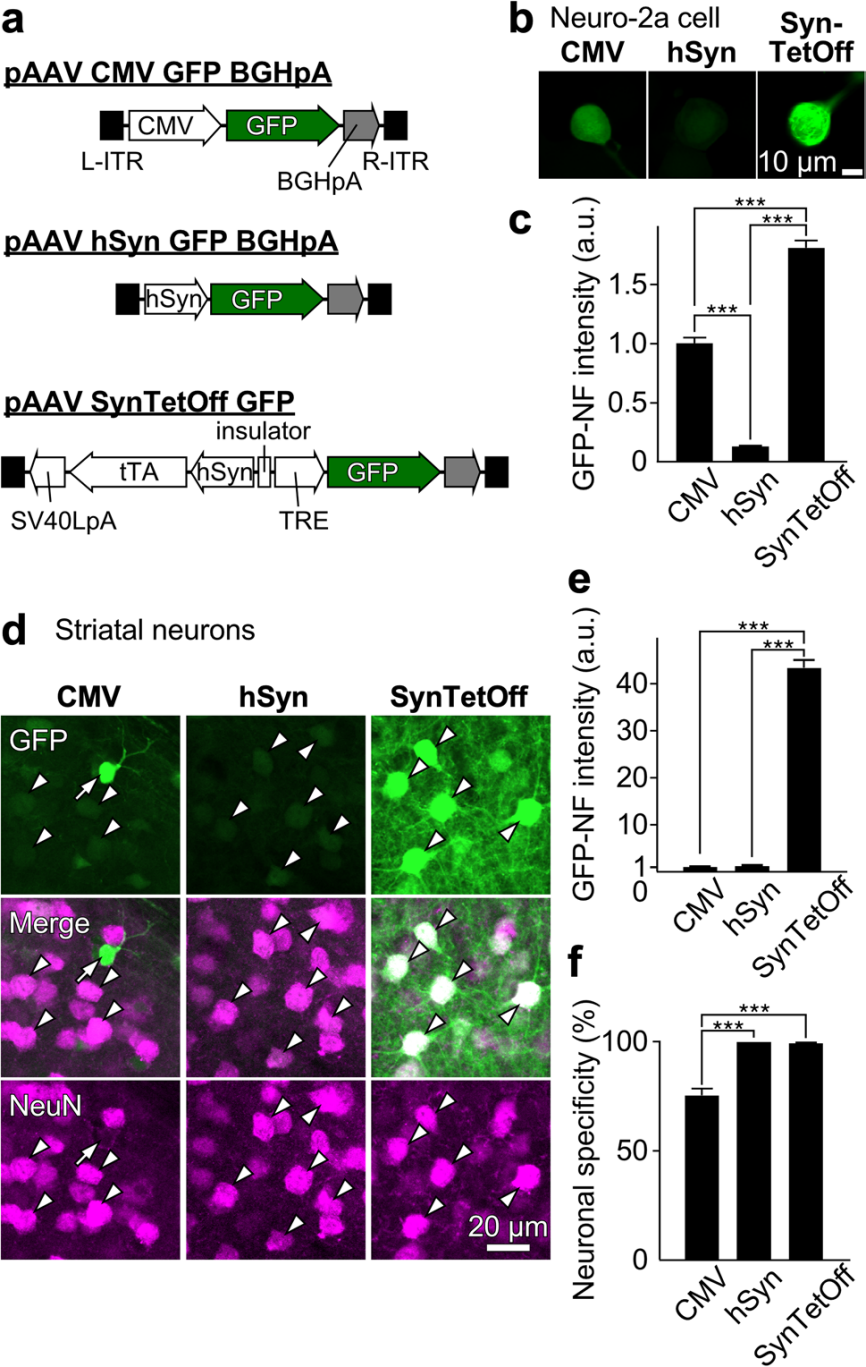
Neuron-specific high-level transgene expression with the AAV-SynTetOff vector. **a** Construction of the vector plasmids, pAAV-CMV-GFP-BGHpA, pAAV-hSyn-GFP-BGHpA, and pAAV-SynTetOff-GFP. **b** GFP native fluorescence (NF) by infection of the AAV-SynTetOff-GFP and control vectors in Neuro-2A cells in vitro. **c** Gene-transduction efficiency of the AAV-SynTetOff-GFP and control vectors. The GFP-NF intensity of cells infected with AAV-CMV-GFP-BGHpA is normalized to 1 arbitrary unit (a.u.). **d** GFP expression (green) in neostriatal neurons by injection of the AAV-SynTetOff-GFP and two control vectors with immunolabeling for neuron-specific nuclear protein (NeuN, magenta). Arrowheads indicate GFP-expressing neurons, while an arrow indicates a NeuN-negative putative glial cell. **e** GFP-NF intensities in neostriatal neurons. The mean GFP-NF intensity with AAV-CMV-GFP-BGHpA is normalized to 1. AAV-SynTetOff-GFP transduces much stronger GFP expression in neurons than AAV-CMV-GFP-BGHpA and AAV-hSyn-GFP-BGHpA (factors of 43.3 and 34.3, respectively). **f** Specificities of GFP expression in neostriatal neurons. AAV2/1-hSyn-GFP-BGHpA and AAV-SynTetOff-GFP display specific expression in neurons, while the expression of GFP with AAV-CMV-GFP-BGHpA is not neuron-specific. Error bars, ± standard error of the mean (s.e.m.). ***P < 0.001, Tukey’s multiple-comparison test after one-way analysis of variance (ANOVA). Modified from Sohn et al. (2017)

Recombinant AAV vectors are now widely applied for neuroscience due to their high gene delivery efficiency and low toxicity. Although the human synapsin I (hSyn) promoter-driven gene expression guarantees neuron-selective labeling of both glutamatergic and GABAergic cells, its expression level is insufficient compared with the control of ubiquitous promotors such as the human cytomegalovirus (CMV) and CMV early enhancer/chicken β actin (CAG) promoters (Lukashchuk et al. 2016). To achieve both neuron selectivity and efficiency of gene transduction, we developed a hybrid type AAV platform, AAV-SynTetOff (Sohn et al. 2017); an improved version of tetracycline-controlled transactivator (tTAad) is driven by the hSyn promoter for neuron-selective expression, while a reporter gene is strongly expressed under the tetracycline-responsive element (TRE) promoter to which tTAad binds (Fig. 3a). This AAV-SynTetOff system induces high-level transgene expression selectively in neurons. The expression level in cultured Neuro-2a cells in vitro was approximately 2- and 15-fold higher with the AAV-SynTetOff system than with the conventional AAV vectors driven by the CMV and hSyn promotors, respectively (Fig. 3b, c). Moreover, this AAV vector implements high-level gene transduction in vivo as well (Fig. 3d): the native fluorescence intensity of green fluorescent protein (GFP) was measured in neostriatal neurons infected by the AAV-SynTetOff-GFP vector, showing that this platform allows 34–43 times higher transduction efficiency than the conventional AAV vectors (Fig. 3e). As expected, the transgene was expressed selectively in neurons (Fig. 3f). The promising high-level transgene expression by AAV-SynTetOff-derived vectors can be applied for deep brain imaging with fluorescent proteins (Furuta et al. 2022), bioluminescence (Iwano et al. 2018) or biosensors (Jones-Tabah et al. 2020). In addition, combined with Cre-driver mouse lines, injection of an AAV-SynTetOff vector equipped with *lox* sequences recognized by Cre recombinase can strongly label particular cell populations (Sohn et al. 2016, 2017; Furuta et al. 2022). These technical advances can promote the exploration of neuronal morphology and neural network architecture.

## Spatially selective subcellular configuration of cortical synapses

A single-cell axonal ramification of a presynaptic neuron restrains the encounter with postsynaptic neurons. Corticocortical (Veinante and Deschênes 2003; Rockland 2020; Peng et al. 2021) and thalamocortical (Arnold et al. 2001; Furuta et al. 2009, 2011; Kuramoto et al. 2009, 2015, 2017; Ohno et al. 2012; Nakamura et al. 2015) excitatory efferents as well as GABAergic interneuron axons (Markram et al. 2004; Ascoli et al. 2008; Uematsu et al. 2008; Kubota 2014; Feldmeyer et al. 2018; Gouwens et al. 2020) have preferential laminar (tangential) and columnar (vertical) targets in the neocortex. In addition, postsynaptic neuron dendrites can uniquely spread: for example, each pyramidal cell has a thick, vertically oriented apical dendrite as well as several basal dendrites around its soma. Using an AAV-SynTetOff vector injected into VIP-Cre mice, we showed that L2/3 VIP^+^ neurons in S1BF resemble pyramidal cells in somatodendritic morphology (Sohn et al. 2016, 2017). The dendritic morphologies of pyramidal cells and L2/3 VIP^+^ neurons in S1BF indicate that they are strongly affected by afferents in L1, where top-down feedback signals from the frontal cortices abundantly terminate (Veinante and Deschênes 2003; Lee et al. 2013; Manita et al. 2015). Thus, we can suppose the synaptic connectivity to some extent from the information of the axonal ramification and somatodendritic morphology.

Moreover, presynaptic neurons can show subcellular target preference in postsynaptic neurons. In particular, individual cell types of GABAergic neurons in the neocortex display unique innervation patterns on the subcellular somatodendritic domains of pyramidal cells, such as somata, dendritic shafts, dendritic spines, and axon initial segments (Somogyi et al. 1998; Thomson and Bannister 2003; Jiang et al. 2013; Kubota 2014; Kubota et al. 2015, 2016). PV^+^ and SOM^+^ neurons preferentially target the perisomatic and dendritic parts in pyramidal cells, respectively, uniquely affecting the pyramidal cell activity (Gentet et al. 2012; Lee et al. 2012; Lovett-Barron et al. 2012; Kamigaki and Dan 2017; Abbas et al. 2018; Xu et al. 2019). For instance, in the visual cortex, PV^+^ and SOM^+^ neurons show divisive and subtractive effects on pyramidal cell responses to visual stimuli (Wilson et al. 2012): the relatively uniform suppression by SOM^+^ cells leads to sharpening the direction selectivity, whereas the non-uniform inhibition by PV^+^ neurons results in proportional gain reduction. Although both inhibit the pyramidal cell excitability, the subcellular localization of GABAergic synapses may cause presynaptic cell type-specific modulation of the postsynaptic cell activity.

In addition, GABAergic neurons form reciprocal synaptic connections with each other, and the inhibitory network displays selective cell-to-cell connectivity based on presynaptic and postsynaptic cell types (Pfeffer et al. 2013; Jiang et al. 2015; Karnani et al. 2016). For example, VIP^+^ cells strongly innervate SOM^+^ and PV^+^ neurons (Pfeffer et al. 2013; Pi et al. 2013; Kepecs and Fishell 2014), potentiating the excitability of pyramidal cells via inhibition of other GABAergic cell types. This disinhibition of pyramidal cell activity by VIP^+^ cells is observable throughout the neocortex, such as the somatosensory (Lee et al. 2013; Karnani et al. 2016), auditory (Pi et al. 2013), visual (Fu et al. 2014; Zhang et al. 2014) and medial prefrontal cortices (Pi et al. 2013; Lee et al. 2019). VIP^+^ neurons in the sensory cortices are recruited by feedback afferents derived from the motor-associated frontal cortices (Lee et al. 2013; Zhang et al. 2014), and they are in fact activated during animal’s movement (Fu et al. 2014; Yu et al. 2019). This functional property of VIP^+^ neurons indicates that top-down motor information may modulate the sensitivity of pyramidal cell responses to sensory stimuli via VIP^+^ neuron-mediated disinhibition. Therefore, the regulation of VIP^+^ cell activity in the sensory cortices is of particular importance for sensory perception.

**Fig. 4:**
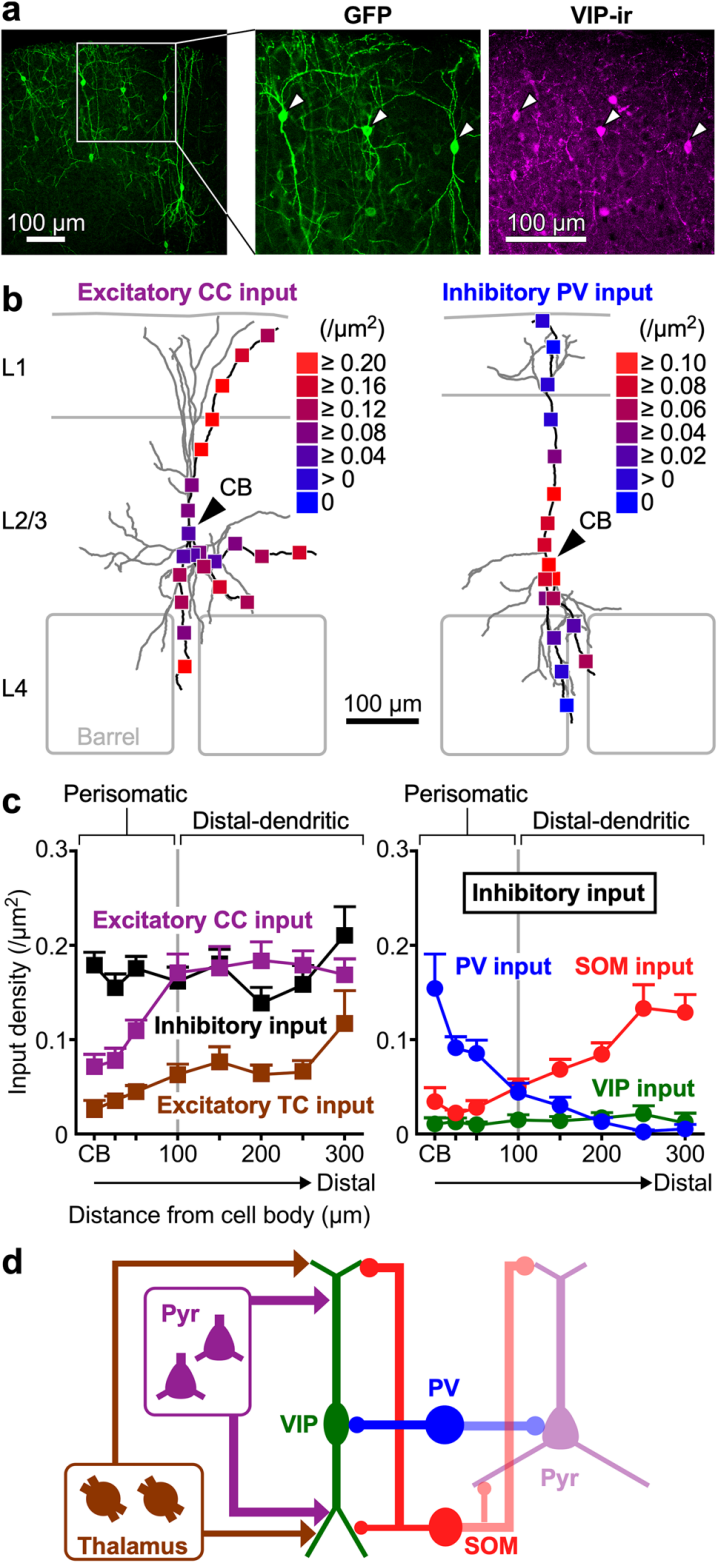
Spatially selective subcellular configuration of synapses on VIP^+^ neurons. **a** Somatodendritic visualization of VIP^+^ neurons with the injection of an AAV-SynTetOff vector into a VIP-Cre mouse. The AAV vector contains double-floxed inverted open reading frame (DIO) for Cre-dependent expression of GFP. Somatodendritic morphology is clearly visualized compared with VIP-immunoreactivity (ir). Arrowheads indicate GFP-expressing VIP^+^ neurons. **b** Densities per surface area (µm^2^) on VIP^+^ neurons of excitatory corticocortical (CC) synapses (left) and inhibitory synapses derived from PV^+^ neurons (right). CB, cell body. **c** Densities (mean ± s.e.m.) of excitatory CC, thalamocortical (TC) and inhibitory synapses along the somatodendritic axis of VIP^+^ neurons (left). Inhibitory synaptic densities originating from PV^+^, SOM^+^ and VIP^+^ neurons are separately quantified (right), showing perisomatic (< 100 µm from CB) and distal-dendritic (≥ 100 µm) targeting of PV^+^ and SOM^+^ neurons. **d** Schematic diagram summarizing subcellular distribution of synaptic inputs to VIP^+^ neurons. Pyr, pyramidal cell. Modified from Sohn et al. (2016)

Electrophysiological recordings have confirmed both excitatory and inhibitory innervation of VIP^+^ neurons (Porter et al. 1998; Rozov et al. 2001; Lee et al. 2013; Pfeffer et al. 2013), and perisomatic synapses on VIP^+^ cells have been morphologically observed with immunohistochemical labeling for VIP (Staiger et al. 1996, 1997, 2002). In the sensory cortices, electron microscopy demonstrated that synapses on VIP^+^ neuron somata were mostly symmetric (Hajós and Zilles 1988), suggestive of GABAergic inputs: indeed, PV^+^ axons form abundant synapses on the somatic region of VIP^+^ cells (Staiger et al. 1997, 2002). However, the electrophysiologically reported postsynaptic events in VIP^+^ neurons evoked by feedback excitatory and SOM^+^ inhibitory afferents (Lee et al. 2013; Pfeffer et al. 2013) had not been fully supported by morphological evidence, probably due to insufficient visualization of vertically elongated VIP^+^ neuron dendrites with immunolabeling for VIP (Fig. 4a). We then applied genetic labeling using an AAV-SynTetOff vector combined with the VIP-Cre mouse line instead of immunostaining (Fig. 4a), and systematically quantified the presynaptic cell type-specific innervation patterns (Sohn et al. 2016) (Fig. 4b). Complete reconstruction of L2/3 VIP^+^ neuron dendrites in S1BF displayed thick-tufted dendrites in L1. Excitatory synapses on VIP^+^ neurons were preferentially formed in the distal dendrites of L2/3 VIP^+^ neurons (Fig. 4b,c), and, in particular, corticocortical glutamatergic axons strongly targeted the distal-dendritic compartment in L1. This morphological investigation of synaptic contacts in VIP^+^ neurons including their distal dendrites could explain the strong effect of top-down corticocortical afferents on VIP^+^ neuron activity (Lee et al. 2013; Zhang et al. 2014).

GABAergic [vesicular GABA transporter-positive (VGAT^+^)] inhibitory innervation showed seemingly uniform distribution in the somatodendritic axis of VIP^+^ neurons (Fig. 4c, left). However, GABAergic subclasses displayed unique subcellular layouts of synapses in VIP^+^ neurons (Fig. 4c, right). PV^+^ neurons preferentially targeted the perisomatic compartment (within 100 µm from somata) of VIP^+^ cells (Fig. 4b, c), consistent with previous reports (Staiger et al. 1997, 2002). By contrast, SOM^+^ inhibitory synapses were observable largely in the distal-dendritic compartment (≥ 100 µm from somata) (Fig. 4c, right), and unidentified GABAergic cells, probably L1 interneurons or neurogliaform cells (Rudy et al. 2011; Jiang et al. 2013; Lee et al. 2015), were assumed to innervate the distal-dendritic compartment in the same manner. This compartmentalized subcellular configuration of GABAergic synapses (Fig. 4d) indicates an inhibitory division of labor in which VIP^+^ neuron excitability is regulated differently between the perisomatic and distal-dendritic compartments. This presynaptic cell type-dependent preference of subcellular targets corresponds to different postsynaptic properties, such as action potential backpropagation and calcium dynamics, between proximal (< 100 µm) and distal (> 100 µm) dendrites of GABAergic cells due to the presence of *I*_A_-type potassium currents (Goldberg et al. 2003). Observation at a subcellular spatial resolution with genetic somatodendritic labeling, therefore, revealed presynaptic cell type-dependent compartments in L2/3 VIP^+^ neurons.

The cell type-specific subcellular distribution of synapses may be a fundamental principle for activity regulation of single neurons. Indeed, the subcellular innervation pattern of PV^+^ neurons also depends on presynaptic cell characteristics (Kameda et al. 2012; Hioki et al. 2013, 2018): we generated a BAC transgenic mouse line in which PV^+^ somatodendritic membrane is selectively labeled with GFP, and described the subcellular layout of synaptic inputs to PV^+^ neurons in S1BF. The somatic compartment of PV^+^ neurons was strongly innervated by VIP^+^ and cholecystokinin^+^ neurons, while PV^+^ reciprocal synaptic connections as well as glutamatergic inputs were formed predominantly in their dendritic compartment. These morphological evidences support the function of VIP^+^ neurons as an upstream element for the disinhibitory circuit in the neocortex (Pfeffer et al. 2013; Pi et al. 2013; Kepecs and Fishell 2014) as well as a presynaptic source directly inhibiting pyramidal cell dendrites (Zhou et al. 2017). In addition to cell type-specific unique laminar and columnar spreads of dendrites and axons, these observations at a subcellular spatial resolution demonstrate non-uniform synaptic configuration in the somatodendritic structures of both pyramidal cells and GABAergic neurons. This “spatial selectivity” of synaptic connections between presynaptic and postsynaptic neurons may cause unique functions of distinct neuronal subpopulations.

## Temporally selective reconfiguration of two types of excitatory synapses

**Fig. 5:**
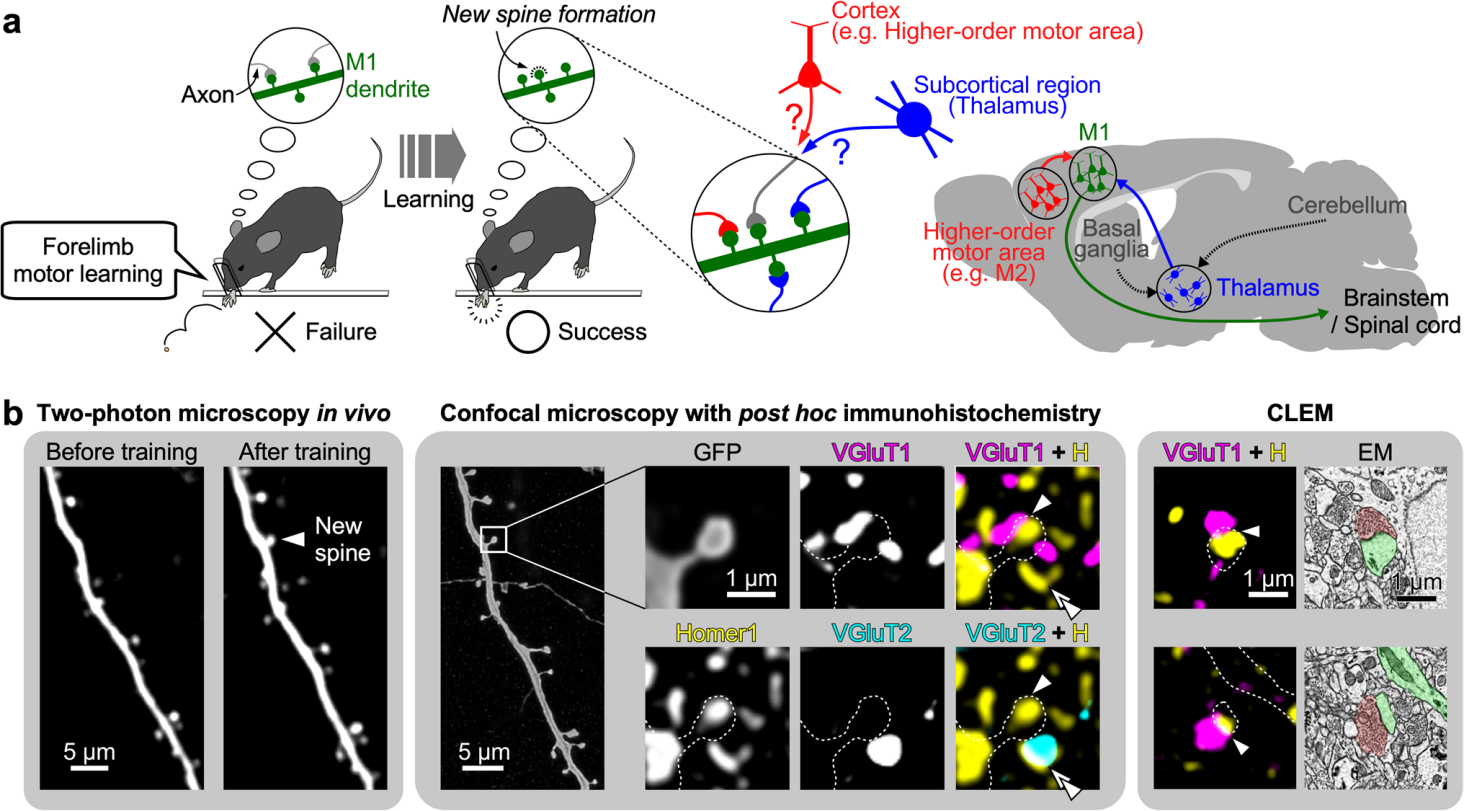
Correlated two-photon, confocal and electron microscopy for identification of the presynaptic origin of inputs to new spines formed during learning. **a** New spine formation in the apical tuft of Thy1-GFP-M mice during a forelimb reaching task. Spinogenesis in M1 coincides with motor skill improvement, but the origin of inputs to the newly formed spines remained to be identified. Both corticocortical and thalamocortical fibers can terminate on the apical tuft. **b** Two-photon microscopy in vivo followed by post hoc immunohistochemistry for presynaptic (VGluTs) and postsynaptic (Homer1) markers. A newly formed spine is observed in the quadruple fluorescence image. Arrowheads indicate a putative corticocortical (VGluT1^+^) synaptic input site. Double-arrowheads indicate a thalamocortical (VGluT2^+^) axon terminal close to the new spine that innervates a different target. Electron microscopy (EM, right) confirms synaptic structure at the putative synaptic input sites indicated by arrowheads. Modified from Sohn et al. (2022)

The “snapshots” of anatomical diagrams in fixed brain tissues show the spatial selectivity of synaptic wirings. Neural networks, however, can be rearranged, and the synaptic dynamics are thought to underlie animal’s learning. Since excitatory synapses in pyramidal cells are typically formed on dendritic spines, the spine plasticity including formation and elimination reflects synaptic dynamics in the neocortex. Two-photon time-lapse imaging in vivo combined with genetic fluorescence labeling allows long-term observation of the spine turnover in the neocortex (Grutzendler et al. 2002; Trachtenberg et al. 2002), demonstrating the correlation of spine plasticity with learning: for instance, motor learning enhances spinogenesis in pyramidal cell dendrites of M1 (Xu et al. 2009; Yang et al. 2009; Fu et al. 2012; Peters et al. 2014; Chen et al. 2015). Although this enhanced spine turnover in M1 should underlie circuit remodeling in motor skill learning, the circuit-based logic behind the change had remained obscure because the description of postsynaptic spine plasticity lacked the information of the presynaptic neuronal partners innervating those spines (Fig. 5a). The upstream partners of M1 originate from both the cortical and subcortical brain regions such as the higher order motor areas and the motor-related thalamic nuclei (Kuramoto et al. 2009, 2015; Hooks et al. 2013; Kaneko 2013; Ueta et al. 2014; Shigematsu et al. 2016; Kawaguchi 2017; Hasegawa et al. 2020) (Fig. 5a). The temporal principle for the circuit remodeling in learning, therefore, requires identification of the presynaptic origin of inputs to the learning-associated postsynaptic spines.

**Fig. 6:**
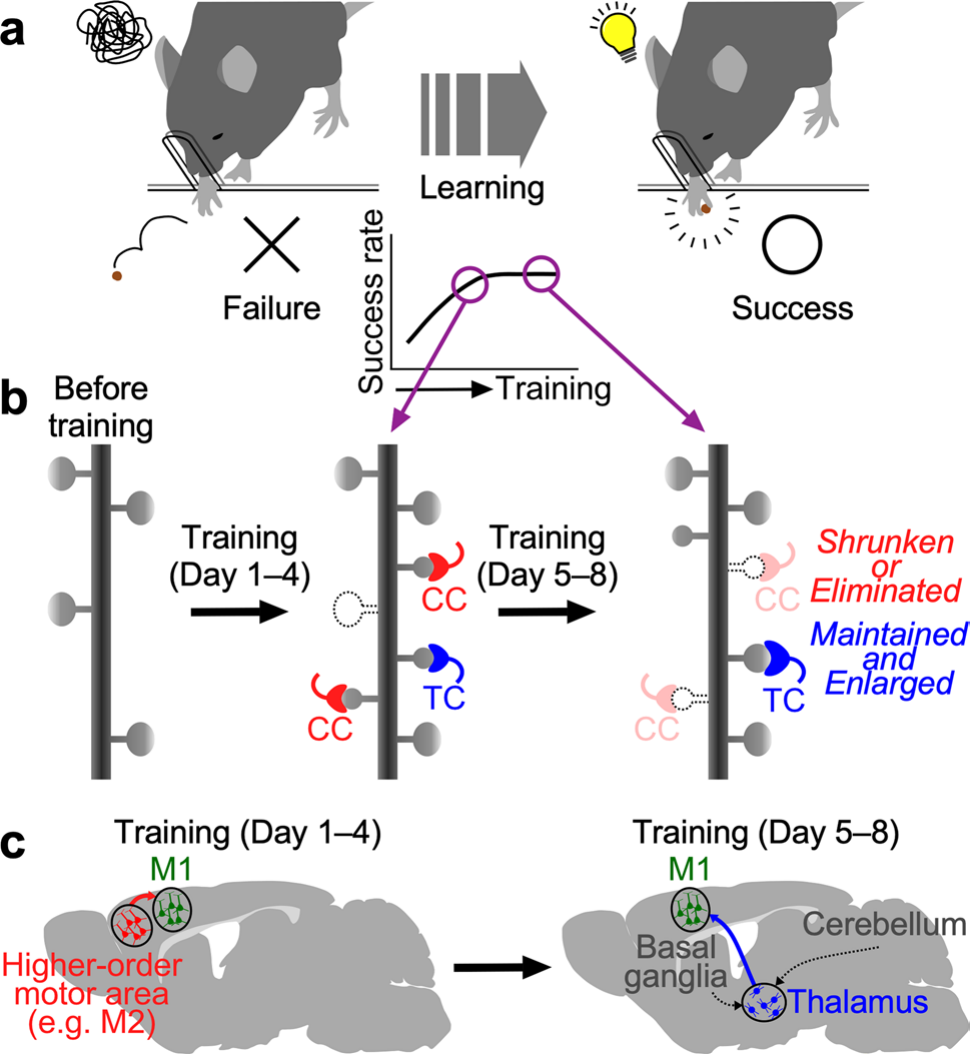
Temporally selective synaptic reconfiguration and circuit remodeling underlying behavior modification. **a** Behavioral improvement in skill learning. Rapid increase in successful reaches is followed by a slowdown in growth. **b** Schematic diagram of spine dynamics in M1 during motor learning. New spines formed during initial 4 days of learning are largely innervated by corticocortical (CC) axons. These corticocortically innervated new spines are mostly pruned during the subsequent 4 days, while new spines innervated by thalamocortical (TC) neurons are sustained and enlarged. **c** Circuit remodeling suggested by synaptic dynamics in M1. Cognitively demanding processes during the early phase of learning involve top-down corticocortical inputs, and the acquired motor skill may be stored long term at thalamocortical innervation sites. Modified from Sohn et al. (2022)

We solved this problem with a combination of experimental techniques, including in vivo two-photon imaging, post hoc immunohistochemistry, confocal microscopy with image deconvolution, and correlated light and electron microscopy (CLEM) (Sohn et al. 2022) (Fig. 5b). We trained Thy1-GFP-M mice (Feng et al. 2000) to reach for single seeds with their preferred forelimbs, and monitored spine dynamics on the apical tufts of GFP-labeled L5 PT pyramidal cells in the forelimb region of M1 under a two-photon microscope. Spine formation and elimination rates were correlated with the skill improvement, consistent with previous studies (Xu et al. 2009; Yang et al. 2009; Wang et al. 2011; Fu et al. 2012; Hayashi-Takagi et al. 2015). After 4 or 8 days of the training and two-photon imaging, we performed post hoc immunohistochemistry for vesicular glutamate transporters (VGluTs; VGluT1 for cortical axon terminals and VGluT2 for thalamic fibers) as well as a postsynaptic marker Homer1. The confocal microscopy at a synaptic spatial resolution successfully determined the origin of inputs to newly formed spines, and synaptic structures were confirmed on these new spines by CLEM. These combined techniques revealed synaptic and circuit rearrangements that underlie behavioral change in animal’s skill learning (Fig. 6). We showed that corticocortical axon terminals predominantly innervated newly formed spines while only a small population of new spines received thalamocortical inputs during initial 4 days of the task training (Fig. 6a, b). During the subsequent 4 days of the task training, however, new spines receiving the corticocortical input were largely pruned while new spines with the thalamocortical synapses survived and grew in size (Fig. 6a, b). Thus, the correlated two-photon, confocal and electron microscopy revealed presynaptic cell type-dependent “temporal selectivity” of synaptic connections during learning: profuse and transient corticocortical synaptogenesis versus fewer but longer-lasting formation of thalamocortical synapses (Fig. 6b).

This input type-dependent rewiring process may explain the circuit-based mechanism for transition from attentive to subliminal motor skill improvement (Fig. 6c). New thalamocortically innervated spines were likely to appear in the dendritic segments with new corticocortically innervated spines, and the active spine formation preceded thalamocortical synaptogenesis (Sohn et al. 2022). These spatial and temporal features of new spines in the dendritic segments may explain branch-specific synaptic plasticity during learning (Yang et al. 2014; Cichon and Gan 2015; Xu et al. 2023). L1 of M1 is strongly innervated by the higher order motor area such as M2 (Ueta et al. 2014; Hasegawa et al. 2020), which is responsible for goal-directed behavior and motor planning (Guo et al. 2014; Li et al. 2015; Allen et al. 2017), and its neuronal ensemble activity can be modified by motor learning (Cao et al. 2015; Makino et al. 2017). The motor thalamic nuclei also project to M1, mediating the afferents from the subcortical regions such as the basal ganglia and the cerebellum (Kuramoto et al. 2011), which are associated with automatic and habitual movement. These functions of the presynaptic cells suggest that “ephemeral” corticocortical synapses that appear only during the learning asymptote might provide learning-associated conditioning of the targeted dendritic segments; these teaching contacts may update internal programs for mnemonic transfer to “immortal” thalamocortical synapses generated in the corticocortically supervised segments. This presynaptic cell type-selective reconfiguration may underlie transition from cognitively demanding efforts to subcortically driven automatic motor control.

The unique contribution of corticofugal and thalamofugal pathways to motor learning and execution was also reported in the striatum. Motor learning requires dorsolateral striatum (DLS)-projecting cortical neurons, while DLS-projecting thalamic neurons are essential for executing consolidated motor skills (Wolff et al. 2022). As shown in the striatum, cortical and thalamic afferents in M1 might play distinct roles in motor learning and memory. Indeed, chemogenetic blockade of corticocortical inputs from M2 impaired skill improvement as well as synaptogenesis in M1 (Sohn et al. 2022). On the other hand, daily blockade of thalamocortical inputs seemingly did not directly affect task performance, but thalamic inactivation hindered the enlargement of thalamocortically innervated spines and, instead, enhanced the maturation of corticocortically innervated spines. This compensation by corticocortical synapses may explain the intact performance under thalamocortical silencing. In addition, daily chemogenetic blockade of corticocortical inputs interferes with skill improvement in the early phase of the skill learning, whereas temporary thalamocortical inactivation after motor skill learning disrupted the task performance of the expert mice (Sohn et al. 2022). These inequivalent programs of synaptic dynamics supervised by two distinct afferents suggest that there are two classes of synapses—those specific to learning and those specific to memory. This overturns the widely held assumption that single synapses must necessarily mediate both learning and memory. Since the neocortex is a repeating lattice with presumed common principles, it can be expected that neural circuit-dependent “temporal selectivity” of synaptic reconfiguration we reported in this study (Sohn et al. 2022) may eventually apply to other cortical areas and learning behaviors.

## Perspectives

To complete the whole-brain wiring diagram is an extremely challenging project. As mentioned above, the subcellular input patterns have been reported in pyramidal, VIP^+^ and PV^+^ cells, and the standard neural circuit has been investigated based on the traditional cell classification. With the advent of large-scale transcriptomics, however, the detailed taxonomy of cortical neurons has been promoted, revealing substantially more diverse cell subtypes than the traditional classification (Zeisel et al. 2015; Tasic et al. 2016; Smith et al. 2019; Gouwens et al. 2020; Sun et al. 2021). The detailed classification will deepen the characterization of the intrinsic morphological and electrophysiological properties as well as the connectivity among these individual subtypes. For instance, SOM^+^ neurons (Tremblay et al. 2016; Nigro et al. 2018; Smith et al. 2019) and L1 interneurons (Schuman et al. 2019; Meng et al. 2023) can be subdivided particularly by their axonal morphologies, and the convergent inputs to these GABAergic neurons have not been fully investigated anatomically. In addition to the analysis of synaptic convergence, the brain network diagram requires the quantitative information of divergent neuronal outputs to postsynaptic targets (Kuramoto et al. 2022). These synaptic wirings should be analyzed simultaneously with the reconstruction of their axonal morphologies, requiring a multi-scale imaging approach with various spatial—from cellular to synaptic—resolutions. CLEM is one of the anticipated potential strategies, and the technologies for imaging (Kasthuri et al. 2015; Phelps et al. 2021) and automatic dense segmentation (Turner et al. 2022) have been improved recently. CLEM with genetic fluorescence labeling and en bloc brain imaging with tissue-clearing methods that preserve ultramicrostructure (Hama et al. 2015; Furuta et al. 2022; Yamauchi et al. 2022a, 2022b) can supply the information of the exact molecular profiles of neurons to monochromatic image data from electron microscopy. Further methodological advances that systematize and semi-automate this laborious project would promote the cell type-specific description of brain-wide synaptic networks.

Although our description of synaptic dynamics in the apical tuft of L5 PT pyramidal cells offers a circuit-based mechanism for learning, there are still several problems to solve in order to reveal the whole picture of the circuit remodeling. First, we could not identify the origin of inputs to eliminated spines due to the technical limitation of post hoc immunohistochemistry, even though the spine elimination rate also increased during learning. Determination of the presynaptic characteristics of disappearing synapses requires both genetically encodable markers for cortical and thalamic inputs and in vivo imaging devices with a much higher spatial resolution than current two-photon microscopes. Second, the principle of synaptic dynamics in the perisomatic basal dendrites can differ from that in the apical tufts. The turnover of dendritic spines is active in the apical tufts of L2/3 pyramidal cells, while the basal dendrites are relatively stable during learning (Chen et al. 2015). Indeed, the apical tufts of L5 pyramidal cells have a unique property of plasticity that does not occur in the basal dendrites, exhibiting long-term synaptic potentiation induced by unpaired low-frequency stimulation (Sandler et al. 2016). The synaptic dynamics of the perisomatic basal dendrites of L5 PT cells, therefore, may adopt a different rule from those of the apical dendrites. Third, the principle of the circuit remodeling in the other cell types of M1 such as L2/3, L5 IT and L6 neurons remains to be clarified. Although L5 PT neurons are essential for motor learning (Otsuka and Kawaguchi 2021), the other cells can also contribute to learning. Indeed, learning develops the representation of the success/failure outcome in L2/3 cells that affects the activity of L5 PT cells (Levy et al. 2020). In addition, in the prelimbic cortex, L5 IT cells functionally contribute to goal-directed learning through the corticostriatal pathway (Hart et al. 2018). Thus, the complete explanation of the circuit-based mechanism in motor skill learning requires further information on presynaptic cell type-specific synaptic remodeling in all of these cell types.

Forth, L5 PT cells have several projection targets, such as the thalamus, superior colliculus and spinal cord, as well as the pontine nuclei (Oswald et al. 2013). PT cells innervate the ipsilateral pontine nuclei (Kita and Kita 2012) as they can be morphologically identified by retrograde labeling from the pontine nuclei (Hallman et al. 1988), but they are not homogeneous: for instance, upper (L5a) and lower L5 (L5b) PT neurons have a trend to project to the thalamus and the spinal cord, respectively, and a part of L5b PT cells target both of the extratelencephalic regions (Ueta et al. 2013, 2014). These divergent PT cells that command movement via projection to the spinal cord may send the efference copy to the motor thalamic nuclei that receive signals from the basal ganglia and the cerebellum; the functions of corticospinal PT neurons in motor execution and learning can depend on the presence/absence of projection to the thalamus. In particular, corticospinal neurons are located in L5b, where abundant thalamic axon fibers terminate. Therefore, corticospinal PT neurons with axon collaterals in the thalamus may form the thalamocortical loop network. The diversity of PT neurons implies the cell type-specific functions that depend on their axonal morphologies. While we did not consider the diversity of GFP-expressing L5 neurons in the Thy1-eGFP-M mouse line, the significance of PT cell outputs in motor learning would further be clarified through the observation of presynaptic cell type-dependent spine dynamics with determination of the variety of postsynaptic L5 PT neurons based on the axonal targeting.

Last, though there have been accumulated evidences that cortical GABAergic neurons are involved in synaptic dynamics and learning, the holistic picture of inhibitory microcircuit remodeling that underlies learning and memory remains elusive. The aforementioned spatially selective GABAergic networks may allow cell type-specific functions particularly in learning. GABAergic subpopulations uniquely contribute to learning and memory throughout the cerebral cortex, such as the hippocampus (McKay et al. 2013; Lovett-Barron et al. 2014; Stefanelli et al. 2016; Yap et al. 2021), the piriform (Canto-Bustos et al. 2022), motor (Chen et al. 2015; Cichon and Gan 2015; Lee et al. 2022; Ren et al. 2022; Yang et al. 2022), medial prefrontal (Cummings and Clem 2020; Cummings et al. 2022), visual (Makino and Komiyama 2015; Khan et al. 2018; Poort et al. 2022), auditory (Letzkus et al. 2011; Abs et al. 2018; Schroeder et al. 2023) and somatosensory cortices (Zhao et al. 2017). PV^+^, SOM^+^ and VIP^+^ neurons, three major subtypes of GABAergic neurons, uniquely contribute to synaptic plasticity (Canto-Bustos et al. 2022) and a variety of learning tasks (Khan et al. 2018; Lucas and Clem 2018; Cummings and Clem 2020; Giorgi and Marinelli 2021; Cummings et al. 2022; Lee et al. 2022; Poort et al. 2022; Ren et al. 2022; Singh and Topolnik 2023). For instance, PV^+^ neurons are known to participate in plasticity during the developmental critical period (Hensch 2005; Rupert and Shea 2022), and the perisomatic inhibitory subtypes such as cholecystokinin^+^ neurons as well as PV^+^ neurons in the hippocampus display opposing plasticity mechanism that may support the memory consolidation (Yap et al. 2021). On the other hand, network remodeling in learning that coincides with synaptic plasticity in pyramidal cell dendrites (Xu et al. 2009; Yang et al. 2009; Wang et al. 2011; Fu et al. 2012; Sohn et al. 2022) should be strongly affected by dendrite-targeting subpopulations such as SOM^+^ and L1 interneurons. Indeed, cortical SOM^+^ neurons have been reported to play a key role in various learning contexts (Hartung and Letzkus 2021) such as motor (Cichon and Gan 2015; Khan et al. 2018; Lee et al. 2022; Ren et al. 2022) and associative learning (McKay et al. 2013; Lovett-Barron et al. 2014; Makino and Komiyama 2015; Zhao et al. 2017; Cummings and Clem 2020; Cummings et al. 2022) with modification of structural synaptic dynamics in pyramidal cell dendrites (Chen et al. 2015; Yang et al. 2022). Interneurons in L1, where profuse top-down signals arrive, form the GABAergic microcircuit and also function in associative memory (Letzkus et al. 2011; Abs et al. 2018; Schroeder et al. 2023). Further, VIP^+^ neurons offer disinhibition by innervating other GABAergic cell types and, consequently, locally gate or trigger synaptic plasticity (Canto-Bustos et al. 2022) particularly in the initial learning phase (Ren et al. 2022). Thus, GABAergic neuron subclasses uniquely constitute neuronal ensembles that encode memory engrams through learning (Stefanelli et al. 2016; Giorgi and Marinelli 2021; Cummings et al. 2022).

GABA synthesis and transmission are critical for cortical plasticity and learning (Posluszny et al. 2015), and long-term synaptic plasticity is influenced by concomitant changes of GABAergic inputs in vitro (Artola and Singer 1987; Mott and Lewis 1991; Bear et al. 1992; Kanter and Haberly 1993; Brucato et al. 1996; Chapman et al. 1998; Grover and Yan 1999; Hsu et al. 1999; Kotak et al. 2017). Moreover, GABAergic synapses on pyramidal cell dendrites are dynamically modulated during learning in vivo as well (Chen et al. 2011, 2012; Villa et al. 2016). However, the identity of the presynaptic GABAergic subpopulation that directly affects the excitatory long-term plasticity is yet to be completely clarified. L1-targeting GABAergic neurons such as Martinotti cells and L1 interneurons can block the active postsynaptic events in the apical dendrites of neighboring pyramidal cells (Murayama et al. 2009; Palmer et al. 2012). The axonal boutons of SOM^+^ Martinotti cells are rapidly eliminated during motor learning (Chen et al. 2015); in addition, SOM^+^ neuron activation destabilizes dendritic spines in the apical tufts of pyramidal cells. In fact, activity of neuronal PAS domain protein 4 (NPAS4)-expressing SOM^+^ neurons during motor learning regulates synaptic remodeling in pyramidal cells of M1 (Yang et al. 2022). Understanding the cell type-specific contribution of GABAergic neurons, such as SOM^+^ Martinotti cells and L1 interneurons, to circuit remodeling during learning would require not only functional but also spatial, anatomical evidences that these GABAergic cell types in fact innervate the dendrites adjacent to the excitatory synapses with long-term plasticity. Addressing these remaining issues will be conceptually transformative and groundbreaking for a new perspective on the dynamic architecture of neural networks and the mechanism of learning and memory.

## Conclusion

In this review, the author focuses on the spatial and temporal selectivity of cortical synaptic wirings that depends on the characteristics of presynaptic and postsynaptic neurons. The description of wiring diagrams at a subcellular spatial resolution requires cell characterization and tool developments including gene engineering and high-resolution microscopes combined with image processing. These technical advances visualize the spatially selective synaptic configuration in the somatodendritic structure of single neurons as well as laminar and columnar spreads of dendrites and axons. Furthermore, in vivo observation of postsynaptic dynamics combined with presynaptic cell identification unveils the principles of synaptic reconfiguration, indicating circuit-specific function in learning. Extending and integrating our knowledge of the spatial configuration and temporal reconfiguration of cortical networks will shed light on how the neocortex works in various functions. Thus, observation of synaptic dynamics based on the subcellular “connectome” may enable us to depict “functional connectome” that underlies multimodal information processing in the neocortex.

## Data Availability

Not applicable.
